# Mapping of Global Research in Endodontics From 2004 to 2023: A Bibliometric Analysis

**DOI:** 10.7759/cureus.75694

**Published:** 2024-12-14

**Authors:** Abdulmohsen Alfadley, Khalid Alfouzan, Pillai Arun Gopinathan, Ikram Ul Haq, Faisal Ahmed Alrumi, Haitham Ali Alahmari, Saleh Abdulrahman Alhellal, Bijesh Yadav

**Affiliations:** 1 College of Dentistry, King Saud Bin Abdulaziz University of Health Sciences, King Abdullah International Medical Research Centre, Ministry of National Guard Health Affairs, Riyadh, SAU; 2 Department of Population Health, King Abdullah International Medical Research Center, Riyadh, SAU

**Keywords:** bibliometric, dental pulp, endodontics, research productivity, root canals

## Abstract

Endodontics, a branch of dentistry, treats diseases and impairments in tissues within and surrounding the natural teeth. The aim of the study was to analyze the publication trends and key features of endodontic research published over the past 20 years across the globe. The quantitative bibliometric research approach was used to extract the data from the Web of Science database. The search strategy consisted of TC=(“root canal” OR “root canals” OR “Pulp chamber” OR “pulp chambers” OR “pulp cavity” OR endodontic OR endodontics) and selected the publication years from 2004 to 2023. The search focused on articles and reviews and excluded all other types of documents. The study reviewed and extracted the following bibliometric parameters: breakdown of research and citations by year, type of journals, accessibility and types of documents, top publishing journals, countries, institutions, authors, most cited articles, and top keywords. Microsoft Excel (v16), R Foundation, and VOSviewer software (v.1.6.10) were used for data analysis. Our search strategy grasped 23,894 (69%) of the 34,680 documents found by the initial search. The selected articles were cited with an average of 20 citations per article, recording an average annual growth of 8.92%. Two journals, the *Journal of Endodontics *and the *International Endodontic Journal* published about one-fourth (n=5859; 24.52%) of the articles. Brazil produced the majority of the research (n=3,976; 16.64%), while the United States contributed the research with the highest citation impact. The University of São Paulo produced the most number of articles, while the University of Zurich gained maximum citation impact. Six authors from Brazil made up the top 10 most prolific authors, and the 10 most cited articles received an average of 692.30 citations each. The most influential keywords identified were "Endodontics," "Enterococcus faecalis," and "Cone-beam computed tomography." Over the past 20 years, endodontics research has significantly increased. Although the United States generated the most significant research in this area, Brazil leads in research productivity. Two prestigious journals published the majority of the research. Researchers and clinicians in the field of endodontics would benefit from the findings of this study.

## Introduction and background

Endodontics is a dental specialty that focuses on the diagnosis and treatment of diseases and conditions affecting the dental pulp and adjacent tissues [1]. There have been notable developments in clinical practice as the scope of endodontic care changes due to the introduction of novel technology, materials, and techniques [2]. Similarly, remarkable research has been performed in the field of endodontics over the past few decades [3]. A thorough grasp of the evolution of endodontic research over time can offer important insights into new trends, important areas of study, and prospects for further advancement. Understanding the global landscape of research in this field is crucial [4]. A useful method for charting the conceptual framework of a field of study is bibliometric analysis, which entails the quantitative evaluation of scholarly publications [5]. This method assists in identifying significant authors, organizations, and countries, as well as the most referenced study topics over a given time period, by examining the quantity, distribution, and impact of publications [6,7].

According to Alfadley et al., the Scopus database identified 24,313 endodontics papers published between 2001 and 2020, with 62% produced in the second decade. The University of São Paulo and the United States were the most contributing institutions and countries, with Brazilian researcher José F. Siqueira being the most productive author [8]. Another study revealed that the Web of Science (WOS) database indexed 17,879 endodontics-related publications from 2010 to 2022, averaging 1375 documents annually. Brazil emerged as the most productive country, followed by the United States, China, and India. England's research garnered the highest citation impact, with the United States and Italy following closely behind [9]. Lima et al. analyzed the articles on guided endodontics published from 2016 to May 2023 [10]. Khan et al. scrutinized the 3,739 documents published in the *International Endodontic Journal* (IEJ) from 1967 to 2020. The study indicates the periodic evolution, breakdown of geographical contribution, and thematic dispersion [3]. Another study delved into the most prevalent research themes across papers published in IEJ and the *Journal of Endodontics* (JOE) from 1980 to 2019, revealing that cone beam computed tomography emerged as the preferred research area in the study's final decade [11]. The JOE published the majority of the 100 most cited articles in endodontic journals, with the United States emerging as the most productive country. Another study evaluated 877 papers on micro-computed tomography in endodontic research and revealed that 58% of the articles were published from 2015 to 2019 [12].

A study calculated the amount of endodontic research conducted in Malaysia from 2001 to February 2021, with the first author being a Malaysian, and found 119 papers. University Sains Malaysia contributed more than one-third of the articles (n=15) published in IEJ. Alrubaig et al. reported that Saudi Arabia occupied the eighth rank with 3.29% of global endodontics research from 2010 to 2022. The study examined publication growth, collaboration patterns, influential institutions, and popular sources. The study showed a rise in research quality and international collaboration. The study highlighted an increase in national research collaboration, indicating a growth in valuable endodontics research in Saudi Arabia [9].

This study aims to conduct a bibliometric analysis of endodontic research worldwide from 2004 to 2023, providing a comprehensive overview of the field's growth trends, significant contributions, and evolving areas of interest. This analysis will provide an evidence-based viewpoint on the changing character of endodontic research, illuminating its scientific and clinical trends listed in WOS databases. This article’s identification of research gaps and trends will help influence future paths in endodontic research and practice, in addition to educating practitioners and researchers globally.

## Review

Methodology

Search Strategy

**Figure 1:**
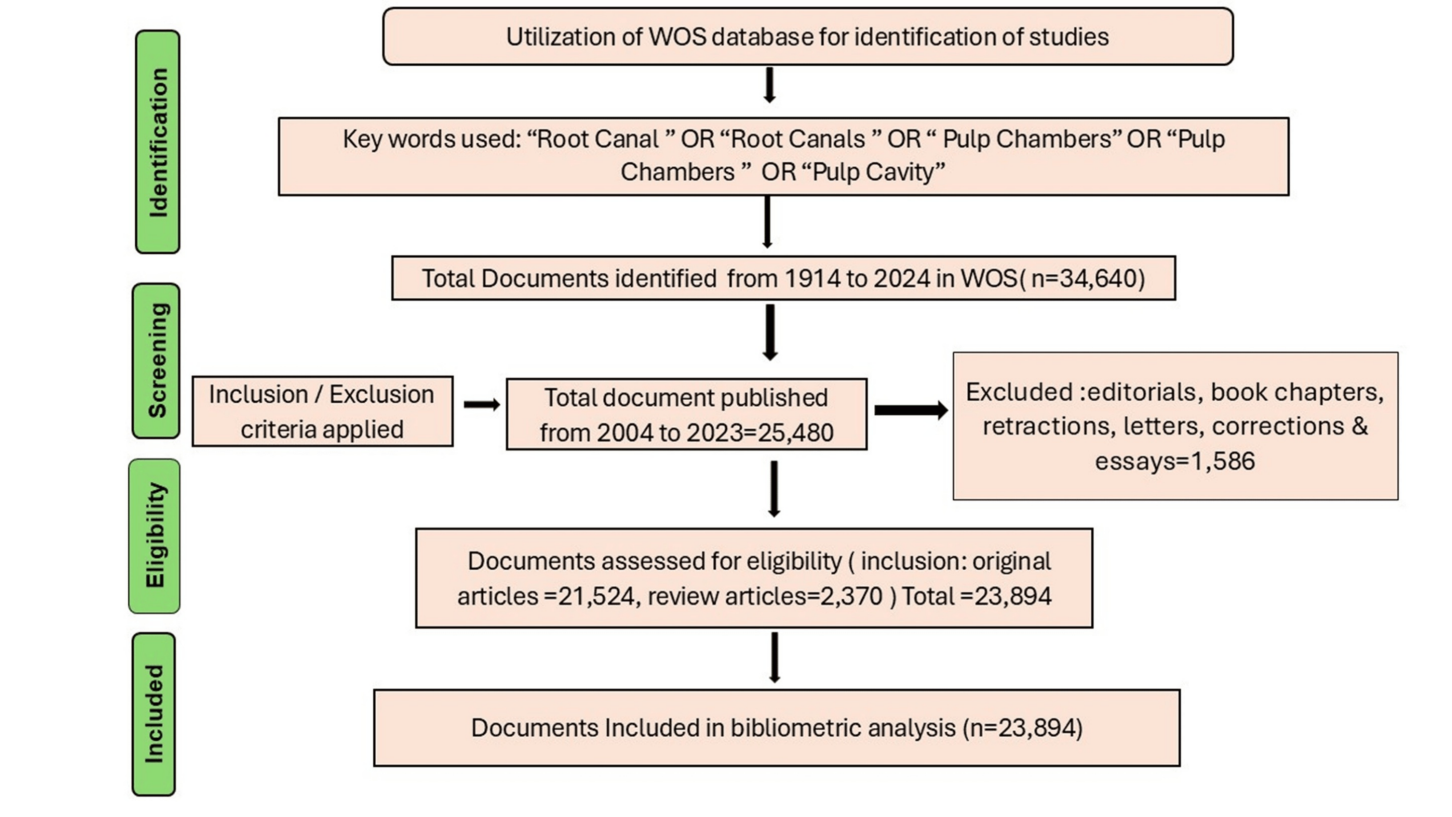
Screening process of articles on Web of Science database WOS: Web of Science database

The study employed the quantitative bibliometric approach to the data set obtained from the Clarivate Analytic WOS database on November 12, 2024 (Figure 1). The search strategy consisted of TC=(“root canal” OR “root canals” OR “Pulp chamber” OR “pulp chambers” OR “pulp cavity” OR endodontic OR endodontics).

Inclusion Criteria

The initial search produced 34,640 documents published between 1914 and 2024. The selected period was 20 years from 2004 to 2023 using the year filter. The search focused on articles and reviews; thus, these two types of documents were only used for analysis.

Exclusion Criteria

All documents published prior to 2004 or after 2023 were excluded. Documents other than articles and reviews, such as meeting abstracts, editorials, letters, book chapters, etc., were also excluded. The methodology is shown in the flow chart (Figure 1).

Bibliometric Indicators

Microsoft Excel (v. 16, Microsoft Corporation, Redmond, USA) and VOSviewer (v. 1.6.10) software were used for data visualization and analysis. The study examined and extracted the following data: breakdown of research by year, including citation impact and annual growth rate; the type of journals; the accessibility modes of articles; the top publishing journals; countries, institutions, and authors; the top 20 keywords; and the 10 most cited articles.

Ethical Consideration

Since the present study used publicly available datasets for analysis and did not utilize human or animal data, institutional review board approval was deemed unnecessary.

Statistical Analysis

The chi-square test was used to compare the percentage of open-access versus subscription-based publications, documents published in dental versus non-dental journals, and clinical versus non-clinical journals. The findings were displayed as a 95% confidence interval (CI) proportional difference, with a p-value less than 0.05 deemed significant. R 4.4 software (R Foundation for Statistical Computing, Vienna, Austria) was used to make the comparison.

Results

Periodic Growth of Articles, Citations, and Annual Growth Rate

Table 1 presents a thorough overview of research productivity over time, including total articles published, total citations, citation impact calculated as the total citations per article, and the annual growth rate in terms of the number of articles published. The 20-year period from 2004 to 2023 observed the publication of 23,894 articles, each with an average of 20.02 citations. The first decade (2004-2013) noticed the publication of about a third of the articles (n=7,572; 32%), while the last decade (2014-2023) saw substantial growth with slightly more than two-thirds of the research (n=16,322; 68%). The first decade observed an average of 35.75 citations per article, while the last decade showed an average of 12.70 citations per article. An average annual growth rate of 8.92% was observed, indicating a steady development of academic output.

**Table 1:** Distribution of articles, citations, citation impact, and annual growth rate by year

Year	Total articles	Total citations	Citation impact	Annual growth rate
2004	434	25,678	59.17	-
2005	491	24,331	49.55	13.13
2006	526	24,893	47.33	7.13
2007	693	27,882	40.23	31.75
2008	752	27,941	37.16	8.51
2009	800	27,252	34.07	6.38
2010	890	32,132	36.10	11.25
2011	904	27,006	29.87	1.57
2012	1,010	27,972	27.70	11.73
2013	1,072	25,612	23.89	6.14
2014	1,223	26,963	22.05	14.09
2015	1,252	27,714	22.14	2.37
2016	1,324	25,957	19.60	5.75
2017	1,368	26,234	19.18	3.32
2018	1,360	22,669	16.67	-0.58
2019	1,537	24,627	16.02	13.01
2020	1,899	21,715	11.43	23.55
2021	2,205	17,018	7.72	16.11
2022	2,077	10,201	4.91	-5.80
2023	2,077	4,530	2.18	0.00
Total/Average*	23,894	478,327	20.02*	8.92*

Comparison of Type of Journals, Accessibility Modes, and Document Type

Table 2 presents the data categories for three variables: journal types, accessibility modes, and document types. The articles published in dental journals dominated both in terms of numbers (n=16,115; 67.44%) and average citations per article (23.99), while the articles published in non-dental journals comparatively gained lower citation impact (11.79). There was a 12% (95% CI: 12.95%-12.45%) significant difference in this category of the journal (p<0.001).

**Table 2:** Comparison of type of journals, accessibility modes and, document type

Categories	Variables	Total articles	Total citations	Citation impact	Difference (95% CI)	p-value
Dental/non-dental journals	Dental journals	16,115	386,601	23.99	12.2 (11.95-12.45)	<0.001
	Non-dental journals	7,779	91,726	11.79		
Accessibility mode	Closed access	14,045	336,362	23.94	9.53 (9.3-9.76)	<0.001
	Open access	9,848	141,965	14.41		
Types of documents	Articles	21,524	395,679	18.38	16.49 (16.14-16.84)	<0.001
	Review articles	2,370	82,648	34.87		

Closed-access articles exhibited a higher ratio (n=14,045; 59%) and acquired 70.32% (n=336,362) of the total citations, resulting in a significantly higher citation impact of 23.94. Conversely, open-access articles have gained a lower level of average citations. There was a 9.3% (95% CI: 9.3%-9.8%) significant difference in this category (p<0.001).

Review articles have a much higher citation impact compared to regular articles. Despite comprising most of the dataset (n=21542; 90%), review articles tend to collect vast volumes of knowledge and, more significantly, receive more references from later studies, as evidenced by their higher citation frequency (34.87 citations per review) compared to regular research articles (18.38 citations per article). The proportion of difference was 16.5% (95% CI: 16.14%-16.84%), which was statistically significant (p<0.001).

Top Publications Sources

The top 10 journals in Table 3 published about 38% (n=8,968) of the articles, which received an average of 31.26 citations each. The JOE stands out with the highest number of articles (n=3786; 15.84%), and these articles achieved a maximum citation impact of 41.24, implying that each of its articles has received over 40 citations. IEJ stood in second rank in terms of the number of articles and citation impact, but it had the highest impact factor (5.4). Despite having fewer articles (599 and 271, respectively), *Clinical Oral Investigations* and *Dental Traumatology* maintain a respectable citation impact (17.60 and 22.63), suggesting that their research had focused on high-impact areas and received frequent citations from scholars.

**Table 3:** Top 10 frequently used journals

Rank	Journal’s name	Impact factor JCR 2023	Total articles	Total citations	Citation impact
1	*Journal of Endodontics* (JOE)	3.5	3786	156144	41.24
2	*International Endodontic Journal* (IEJ)	5.4	2073	75433	36.39
3	Clinical Oral Investigations	3.1	599	10545	17.60
4	Australian Endodontic Journal	1.3	545	4674	8.58
5	Oral Surgery Oral Medicine Oral Pathology Oral Radiology and Endodontology	1.45	442	13700	31.00
6	ENDO - Endodontic Practice Today	No	339	575	1.70
7	BMC Oral Health	2.6	335	3024	9.03
8	Journal of Clinical and Diagnostic Research	0.2	318	1620	5.09
9	Dental Traumatology	2.3	271	6132	22.63
10	Journal of Dentistry	4.8	260	8504	32.71

Top Countries

**Figure 2:**
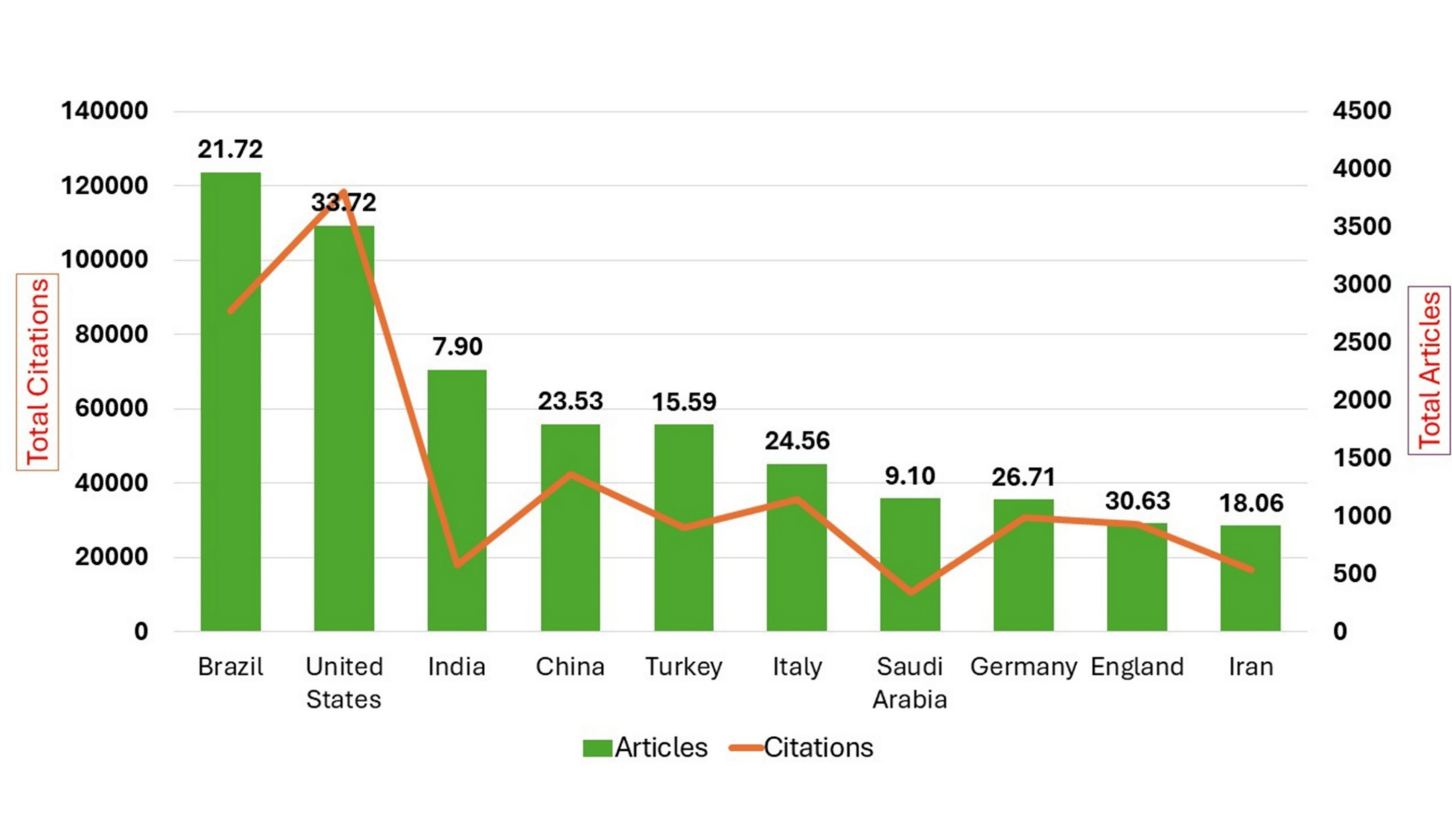
Top 10 productive countries showing distribution of total citations and total articles

Figure 2 and Table 4 present the top 10 productive countries with research productivity and its citation impact. Brazil has produced the utmost quantity of articles (n=3,976; 16.64%), followed by the United States (n=3,514; 14.70%) and India (n=2,270; 9.50%). The United States had the highest citation impact at 33.72, followed by England and Germany with an average of 30.63 and 25.71 citations per article, respectively. India, despite having a large number of articles, had a relatively low citation impact.

**Table 4:** Top 10 productive countries showing citation impact

Rank	Country’s name	Total articles	Total citations	Citation impact
1	Brazil	3976	86349	21.72
2	United States	3514	118487	33.72
3	India	2270	17930	7.90
4	China	1798	42315	23.53
5	Turkey	1793	27948	15.59
6	Italy	1455	35742	24.56
7	Saudi Arabia	1156	10520	9.10
8	Germany	1148	30663	26.71
9	England	941	28826	30.63
10	Iran	923	16673	18.06

Top Institutions

Table 5 ranks academic and research institutions based on the research output and its impact. The University of São Paulo leads in terms of total articles (n=996); São Paulo State University and State University of Campinas stood in the second and third ranks with 678 and 444 articles, respectively. The University of Zurich leads in citation impact (49.55), followed by the University of London and the University of Hong Kong with an average of 36.99 and 36.12 citations per article, respectively. The articles produced by the authors of the Saveeta Institute of Medical Technical Science gained the lowest citation impact among the top 10 institutions. The University of São Paulo and São Paulo State University publish large volumes of research (996 and 678 articles, respectively), and their citation impact (22.46 and 18.45) was moderate.

**Table 5:** Institutional-level analysis

Rank	Institution’s name	Total articles	Total citations	Citation impact
1	University of São Paulo, Brazil	996	22370	22.46
2	São Paulo State University, Brazil	678	12511	18.45
3	State University of Campinas, Brazil	444	12914	29.09
4	University of London, England	407	15053	36.99
5	Saveeta Institute of Medical Technical Science, India	322	3826	11.88
6	King Saud University, Saudi Arabia	296	3840	12.97
7	University of Hong Kong, China	292	10548	36.12
8	Federal University do Rio Grande Do Sul, Brazil	273	4536	16.62
9	University Do Grande Rio, Brazil	272	6685	24.58
10	University of Zurich, Switzerland	255	12634	49.55

Top Researchers

Table 6 lists the top 10 researchers, most of whom were based in Brazil, with 6 out of 10 affiliated with Brazilian institutions. This means that Brazil has a vibrant research community in the area that this data represents. The other researchers came from institutions in the United States, Canada, Australia, and Italy, as evidence of their global influence in endodontic research. With 204 articles, Emmanuel Joao Nogueira Leal da Silva emerged as the top researcher, closely followed by Marco Antonio Hungaro Duarte with 195 articles. Jose F. Siqueira Jr. had the highest average citations per article, with an average of 66.86 citations, demonstrating the high citation impact of his articles. Emmanuel Joao Nogueira Leal da Silva had a relatively lower citation impact (21.19) compared to others, despite having the highest number of total articles.

**Table 6:** Top 10 researchers

Rank	Researcher’s name	Affiliation	Total articles	Total citations	Citation impact
1	Emmanuel Joao Nogueira Leal da Silva	University do Grande Rio, Brazil	204	4323	21.19
2	Marco Antonio Hungaro Duarte	University of Sao Paulo, Brazil	195	4279	21.94
3	Brenda P. F. A. Gomes	State University of Campinas, Brazil	169	5720	33.85
4	Manoel Damia Sousa-Nato	University of Sao Paulo, Brazil	165	4049	24.54
5	Jose F. Siqueira Jr.	University Estacio de Sa, Brazil	142	9494	66.86
6	Franklin R. Tay	Augusta University United States	132	6496	49.21
7	Gianluca Plotino	Catholic University of Rome, Sapienza University, Private practitioner	129	5291	41.02
8	Ove A. Peters	University of Queensland Australia, University of Pacific United States	127	4919	38.73
9	Macro A. Versiani	University Federal Fluminense, Brazil	117	4342	37.11
10	Ya Shen	University of British Columbia Canada	117	5520	47.18

Top Cited Articles

The top 10 most cited articles published between 2004 and 2010 garnered 6923 citations, with an average of 692.30 citations per article, as shown in Table 7. Seventeen authors belonging to eight institutions in five countries contributed to the top 10 most cited articles, and only two authors (Parirokh and Torabinejad) contributed to two articles. A single author penned four articles, while research collaboration resulted in six others. Three journals published these articles, including eight in JOE, one in IEJ, and one in Critical Review of Oral Biology and Medicine. Out of 10 documents, nine were review articles. The authors from the United States contributed to half of the articles, followed by Switzerland and Iran with three and two articles, respectively. The authors from Germany (European Society of Endodontology) and Brazil contributed a single article each. The authors from the University of Texas Health Science Center and the University of Zurich contributed three articles each, followed by two articles jointly contributed by Loma Linda University and Kerman University of Medical Sciences.

**Table 7:** Top 10 most cited articles

Serial No.	Article’s title	Total articles	Citation density by year (rank)
1	Root canal irrigants [13]	1034	54.42 (1)
2	Mineral trioxide aggregate: a comprehensive literature review - part III: clinical applications, drawbacks, and mechanism of action [14]	770	51.33 (2)
3	Current challenges and concepts in the preparation of root canal systems: a review [15]	759	36.14 (5)
4	Quality guidelines for endodontic treatment: consensus report of the European Society of Endodontology [16]	709	37.32 (4)
5	Mineral trioxide aggregate: a comprehensive literature review - part I: chemical, physical, and antibacterial properties [17]	677	45.13 (3)
6	Enterococcus faecalis: its role in root canal treatment failure and current concepts in retreatment [18]	650	34.21 (6)
7	Pathogenesis of apical periodontitis and the causes of endodontic failures [19]	645	30.71 (9)
8	Post placement and restoration of endodontically treated teeth: a literature review [20]	581	27.67 (10)
9	Regenerative endodontics: a review of current status and a call for action [21]	556	30.89 (8)
10	Clinical implications and microbiology of bacterial persistence after treatment procedures [22]	542	31.88 (7)

Top 20 Keywords

Table 8 presents data on the top 20 most occurred keywords generated via VOSviewer software. The occurrences indicate the frequency with which the authors mention the keyword, as well as the overall link strength, which gauges the relevance of the keyword and the strength of its linked text. The keyword "Endodontics" holds the top position with 2,426 occurrences and a link strength of 1151, indicating its high frequency in texts and strong linkage within its context. The keywords “Root canal treatment” (ranked 3rd) and “Root canal” (4th) are noticeable, showing their relevance in discussions about endodontics. Their link strength, at 344 and 447, respectively, indicates that they are moderately well-linked but not the top performers. "Enterococcus faecalis" and "Sodium hypochlorite" rank second and sixth, respectively, with 741 and 632 occurrences. However, their link strengths differ, with *Enterococcus faecalis* having a link strength of 566 and sodium hypochlorite significantly higher at 612, indicating that they are the second most well-liked overall. Regenerative endodontics (ranked 18th) had 297 occurrences and a low link strength of 92, indicating that it is relatively new or emerging in the field of endodontics.

**Table 8:** Top 20 most occurred keywords

Rank	Keyword	Occurrences	Total link strength
1	Endodontics	2426	1151
2	Enterococcus faecalis	741	566
3	Root canal treatment	716	344
4	Root canal	714	447
5	Apical periodontitis	667	356
6	Sodium hypochlorite	632	612
7	Cone-beam computed tomography	607	275
8	Calcium hydroxide	545	455
9	Endodontic treatment	533	229
10	Root canal therapy	495	312
11	Mineral trioxide aggregate	478	144
12	Root canal preparation	429	269
13	Irrigation	348	309
14	Chlorhexidine	343	425
15	Smear layer	307	202
16	Biofilm	304	303
17	Dentin	300	178
18	Regenerative endodontics	297	92
19	Micro-computed tomography	291	124
20	Retreatment	258	117

**Figure 3:**
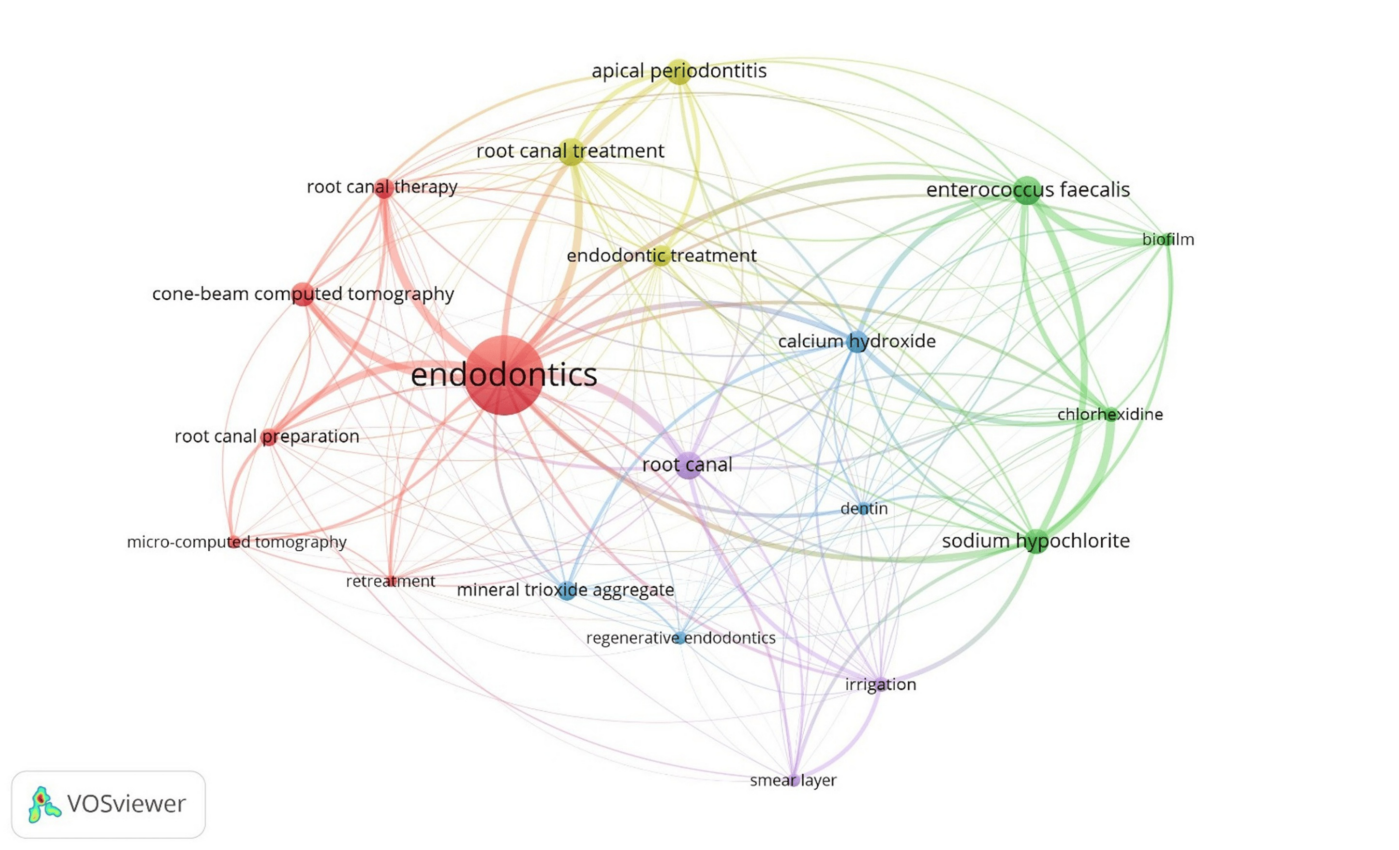
Co-occurrence network of top 20 keywords

Figure 3 shows the visualization network of the top 20 co-occurred keywords generated via VOSviewer software, and these keywords consisted of five clusters. The clustering algorithm looks for keywords that have frequent interactions or connections with one another and groups them together as a cluster. There were six keywords (cone-beam computed tomography, endodontics, micro-computed tomography, retreatment, root canal preparation, root canal therapy) in the first cluster. Among the keywords, four were in the second cluster (biofilm, chlorhexidine, enterococcus faecalis, sodium hypochlorite) and three were in the third cluster (calcium hydroxide, dentin, mineral trioxide aggregate, regenerative endodontics). The fourth cluster had three keywords (apical periodontitis, endodontic treatment, root canal treatment), while the fifth cluster had three keywords (irrigation, root canal, smear layer).

Discussion

The current study quantitatively evaluated the global scenario of scientific and scholarly publications in the subcategory of dentistry and endodontics from 2004 to 2023. The bibliometric assessment provides scholars and practitioners with the prevailing trends and patterns of literature as well as support in determining upcoming research projects [8,23]. Researchers frequently use these studies to assess the state of global research and compare it to the performance of national research [9]. The findings of our study comprised endodontic research over 20 years, with 23,894 articles published between 2004 and 2023 with an average of 20 citations per article. The first decade recorded 32% of articles, while the last decade observed 68% growth. The first decade had 35.75, while the last decade had 12.70 citations per article. Usually, bibliometric studies report that older papers have more time to get citations [24]. Khan et al. examined the 3,739 documents published in the IEJ from 1967 to 2020. Less than one-third (30%) of the records were published in the first 33 years from 1967 to 1999, and 70% of the records were published from 2000 to 2020 [3].

The difference in citation impact between dental and non-dental journals most likely results from the higher volume of research and the emphasis on specialized areas in dental publications. It indicates that dental research published in specific dental journals is focused and frequently cited in contrast to the research published in general and multidisciplinary non-dental journals [25]. Our study reported that articles published in dental research had a larger citation impact, making them more influential compared to articles published in non-dental journals. Higher academic status seems to be associated with closed-access journals, which results in more citations per article, while open-access articles couldn’t have more attention and gained fewer citations per article [5]. Review articles were essential for synthesizing research findings and typically gained much higher citation attention as compared to normal research articles. Fardi et al. reported in their study that among the 100 top-cited articles, the majority (n=17) consisted of review articles, closely followed by 15 case series [4].

JOE stands out as the most influential journal in terms of articles, citations, and citation impact, making it a leading authority in endodontics research, as shown in our study. The IEJ has the highest impact factor, representing extensive visibility and influence in the global dental community. In line with these findings, Lima et al.'s bibliometric study on guided endodontics stated that about one-third of the total articles were published in the JOE and IEJ [10]. Another study on top-cited articles published in endodontic journals stated that more than half of the articles (n=54) were published in JOE [4]. Another study measured the research on micro-computed tomography in endodontic research and revealed that nearly 50% of the research was published in the JOE and the IEJ [12]. A bibliometric study on Saudi Arabian contributions to endodontic research also highlighted that the JOE was a preferred publication source, followed by the Saudi Dental Journal [9].

Our study revealed that the United States had a strong position in the international academic world, as evidenced by its dominance in both overall citations and citation impact. Although Brazil produced the most publications, its citation impact is only moderate, suggesting that its research may not be as influential as that of other countries like the United States, England, or Germany. Brazil (15.90%) emerged as the most productive country in endodontic research from 2010 to 2022, followed by the United States (12.33%), but England's research gained the highest citation impact, followed by the United States and Italy. The bibliometric analysis of the 3,739 documents published in the IEJ revealed that the majority of the articles were contributed by authors from England, with Brazil and the United States following closely behind. The research from Sweden received the highest average citations, with the Netherlands following closely behind [3]. Fardi et al. measured the top-cited articles published in endodontic journals, revealing that authors from 20 countries produced these articles, with the United States contributing 54% and Sweden contributing 13% [4].

Our study highlighted that the University of São Paulo had been the top institution in terms of total articles and citations, reflecting its significant contribution to research output, though its citation impact is lower compared to other leading institutions. The University of Zurich and the University of London stand out top in citation impact. This indicates that these institutions publish highly influential and widely cited research. A study focusing on papers published in the IEJ from 1967 to 2020 stated that the University of São Paulo contributed the greatest number of papers, but the research contributed by the University of Oslo gained the highest citation impact. Ordinola-Zapata et al. examined the most frequently occurring themes published in IEJ and JOE from 1980 to 2019 [11]. This study highlighted the institutions with the highest number of citations in each decade. In the first decade, the University of São Paulo secured the top rank, and in later decades, University College London, Loma Linda University, and the United States Army obtained the top positions [11]. Another study, based on the top-cited articles in endodontics, revealed that Loma Linda University emerged as the most contributing institution [4].

Our study's analysis of top authors revealed that Brazilian researchers were the most productive, both in terms of total articles and citations. Jose F. Siqueira Jr. emerged as the top author in terms of citations and citation impact (66.86 cites/article), closely followed by Franklin R. Tay, a researcher from Augusta University, United States, with fewer articles (n=132) but a relatively high citation impact (49.21 cites/article). Brazil appears to be a center of research in the field of endodontics, with several leading researchers, which indicates a strong academic community and contribution from Brazilian institutions. Alfadley et al. also confirmed this finding in their study on endodontic research published between 2001 and 2020, mentioning José F. Siqueira as the most prolific author [8]. Fardi et al.’s study of 100 top-cited articles on endodontics reveals that M. Torabinejad emerged as a prolific author with 16 articles [4]. In our study, the top 10 most cited articles found that 17 authors from eight institutions in five countries (the United States, Switzerland, Iran, Germany, and Brazil) contributed to these articles, while only two authors (Parirokh and Torabinejad) contributed to two articles. The examination of the top 10 most-cited articles received an average of 692.30 citations, published between 2004 and 2010. Various journals published the articles, nine of which were review articles. The authors from the University of Texas Health Science Center contributed to the majority of the articles. The factors may include research-related capital support, proficiency in English, access to internet databases, and the keywords employed for data retrieval. Few Asian nations were represented in the top 10 cited publications, and factors such as inadequate funding, language barriers, healthcare system deficiencies, and limited access to research data may impede research advancement [25].

The findings of our study regarding the thematic analysis show that “Endodontics” was clearly the central term, with the highest number of occurrences, and had a strong link strength. Topics like “root canal treatment” and “Enterococcus faecalis” were both well-represented in the literature and widely referenced in the endodontic community, making them key areas of focus in the field. Ordinola-Zapata et al. conducted an inspection of the most frequently occurring themes published in IEJ and JOE from 1980 to 2019. Key terms like "canal," "molar," and "periapical lesions" were the most frequently featured in the titles of these journals between 1980 and 1999, and there has been a rise in the number of papers focused on "cone beam computed tomography" during the last 10 years, from 2010 to 2019 [11]. Another study scrutinized the documents published in the IEJ from 1967 to 2020 and reported that “endodontics,” “root canal treatments,” and “calcium hydroxide” had emerged as the topmost research themes [3]. "Endodontic microbiology" (n=17) emerged as the topmost theme in a study of the most cited articles on endodontics, followed by "leakage" (n=15), "canal instrumentation" (n=13), "MTA" (n=13), and "irrigation" (n=12) [4]. Akosy et al. examined the approach to micro-computed tomography in endodontic research and found that "root canal preparation," "root canal anatomy," and "canal filling" were the dominant research themes [12]. Some more specialized or newer terms, like “regenerative endodontics,” had fewer occurrences and lower link strength, indicating they may still be growing in significance. Regenerative endodontics pertains to physiologically oriented techniques aimed at restoring compromised tooth structures, encompassing dentin, root components, and the cellular elements of the dentin-pulp complex. The primary objective of regenerative procedures is to restore pulp function by regenerating it from existing stem cells in the apical tissues or by introducing distant stem cells into the disinfected root canal, thereby creating conditions that promote their differentiation into pulp or pulp-like tissue. Analyzing these findings is crucial for determining their influence on regenerative endodontics in future research and development [24].

The WOS database was selected as the bibliographic data source for the study due to its prominence as a widely utilized resource for bibliometric analysis; also, it has not been used in previous bibliometric studies, particularly in this period from 2004 to 2023. Nevertheless, WOS provides more comprehensive information and enhanced graphics compared to Scopus's citation analysis, and it is likely that WOS was developed with the intention of satisfying users in citation analysis. Moreover, it is acknowledged as the benchmark in publishing bibliometric studies [23].

Limitations

The study excluded certain document types, such as editorials, letters, conference abstracts, and non-English articles. These types of documents might contain insights and contribute to a broader understanding of research trends, but our study focused on documents that usually attract more citations. Articles between 1914 and 2004 were excluded as they were less cited and had poor peer review, while the year 2024 is still progressing. The year span of 2004-2023 was the most productive as far as bibliometric indicators obtained from the WOS database, while further studies are required to go in-depth in the analysis of these articles. The study restricted its search terms to specific keywords such as "root canal," "pulp chamber," and "endodontics," potentially excluding related topics or new research areas that did not align with these keywords, thereby compromising the comprehensive representation of the field.

Also, the study depends exclusively on the WOS-indexed research, which may not include all relevant publications in the field of endodontics. Other databases such as Scopus, PubMed, or Google Scholar could potentially yield different results, limiting the generalizability of the findings. The content coverage of Google Scholar is ambiguous, and the accuracy of results is inconsistent, while citation analysis is overlooked by PubMed [23]. Consequently, research might be undertaken to amalgamate all search databases of Scopus and PubMed for comprehensive coverage of this extensive topic in future studies.

The study primarily relied on citation counts as an indicator of research impact. However, citation counts do not necessarily reflect the quality or clinical relevance of the research, which could lead to an incomplete understanding of the importance of different studies. By recognizing these limitations, future research could assume a broader approach, magnify the scope of data sources, and explore alternative methodologies to provide a more inclusive scenario of endodontic research.

## Conclusions

This bibliometric analysis highlighted the significant developments in endodontic research during the previous 20 years, revealing a substantial increase in research productivity and citation impact. Brazil has become the leader in research output, providing the most papers, while the United States leads in citation impact. JOE and IEJ have widely disseminated most of the research. Significant keywords influencing the field include "endodontics," "enterococcus faecalis," and "cone-beam computed tomography." Researchers and clinicians could benefit from these findings, which can assist in clarifying global endodontic research trends and direct future studies in this area.
